# 568. Comparative Analysis of Cefazolin Target Attainment in Methicillin-Susceptible Staphylococcus aureus (MSSA) Isolates With and Without the Cefazolin Inoculum Effect (CzIE)

**DOI:** 10.1093/ofid/ofaf695.177

**Published:** 2026-01-11

**Authors:** Barbara A Santevecchi, Kathryn DeSear, Nicole Maranchick, Charles Peloquin, William R Miller, Cesar A Arias, Diana Panesso-botero, Samie Rizvi, Cecilia Tran, Nicole M Iovine, Kartikeya Cherabuddi, Venugopalan Veena

**Affiliations:** University of Florida College of Pharmacy, Gainesville, Florida; UF health Shands Hospital, Gainesville, Florida; University of Florida College of Pharmacy, Gainesville, Florida; University of Florida College of Pharmacy, Gainesville, Florida; Houston Methodist Research Institute, Houston, TX; Houston Methodist and Weill Cornell Medical College, Houston, TX; Houston Methodist Hospital, Houston, TX; Houston Methodist Hospital, Houston, TX; Houston Methodist Hospital, Houston, TX; University of Florida, Gainesville, FL; University of South Florida - Tampa General Hospital, Tampa, Florida; University of Florida, College of Pharmacy, Gainesville, Florida

## Abstract

**Background:**

Clinical failures with cefazolin have been linked to the CzIE where increased bacterial inoculum leads to elevated cefazolin minimum inhibitory concentrations (MICs) against MSSA. The role of therapeutic drug monitoring (TDM) in optimizing cefazolin dosing in the context of the CzIE remains unclear. This study aimed to determine the prevalence of the CzIE and compare cefazolin pharmacokinetic/pharmacodynamic (PK/PD) target attainment in MSSA isolates with and without the CzIE.

**Methods:**

A single-center, retrospective review included adults (≥18 years) with MSSA bacteremia admitted between Jun 2021–Dec 2023 who received cefazolin and had steady-state serum cefazolin concentrations. CzIE was defined as a cefazolin MIC ≥16 µg/mL at high inoculum (10⁷ CFU/mL) and ≤8 µg/mL at standard inoculum (10⁵ CFU/mL). Total cefazolin concentrations were adjusted for 80% protein binding. PK/PD targets were 100%*f*T≥MIC and 100%*f*T≥4×MIC (100% free drug time above MIC or 4x MIC, respectively).

**Results:**

Among 51 patients included in the analysis, median age was 61 years (IQR, 39.5–70.5). Common bacteremia sources included endocarditis (n=11, 21.6%), musculoskeletal (n=11, 21.6%), and pneumonia (n=9, 17.6%). Nine isolates (17.6%) exhibited the CzIE and 32 (62.7%) were *blaZ*-positive. Median cefazolin trough was 5.9 µg/mL (IQR, 3.2–11.6) in CzIE infected vs. 4.2 µg/mL (IQR, 2.5–10.4) in patients infected with a non-CzIE isolate. In CzIE isolates, modal MICs were 1 µg/mL at standard inoculum and 16 µg/mL at high inoculum; in non-CzIE, both were 0.5 µg/mL. In the CzIE group at standard inoculum, 100% (n=9) achieved 100%*f*T≥MIC and 66.7% (n=6) achieved 100%*f*T≥4×MIC; at high inoculum, 11.1% (n=1) achieved 100%*f*T≥MIC and none achieved 100%*f*T≥4×MIC. In the non-CzIE group at standard inoculum, 88.1% (n=37) achieved 100%*f*T≥MIC and 73.8% (n=31) achieved 100%*f*T≥4×MIC; at high inoculum 88.1% (n=37) achieved 100%*f*T≥MIC and 61.9% (n=26) achieved 100%*f*T≥4×MIC.

**Conclusion:**

Cefazolin PK/PD target attainment was markedly reduced in patients with CzIE isolates at high inoculum. These findings underscore the potential utility of TDM in detecting suboptimal cefazolin exposure and the need to consider alternative dosing strategies in this setting.

**Disclosures:**

Cesar A. Arias, MD/PhD, UptoDate: Royalties

